# Artificial Intelligence in Otolaryngology—From Demonstrating Capability to Delivering Trustworthy Clinical Impact

**DOI:** 10.1002/wjo2.70187

**Published:** 2026-10-18

**Authors:** Da‐Qing Li

**Affiliations:** ^1^ Department of Otorhinolaryngology–Head and Neck Surgery University of Pennsylvania Perelman School of Medicine Philadelphia Pennsylvania USA

Artificial intelligence (AI) is rapidly transforming otolaryngology–head and neck surgery. Organized by our guest editors, Dr. Waleed M. Abuzeid and Dr. Qintai Yang, this Special Issue brings together 15 contributions that illustrate the remarkable breadth of this evolution, encompassing large language models (LLMs), clinical communication, voice analysis, medical imaging, computational and spatial pathology, predictive modeling, surgical video analysis, ambient documentation, and patient‐centered applications. Collectively, these studies illustrate how AI is moving beyond proof‐of‐concept demonstrations toward increasingly sophisticated clinical applications. At the same time, they remind us that technical performance alone is not sufficient to establish clinical value.

Generative AI and LLMs represent one of the most rapidly expanding areas of AI research. The evaluation of ChatGPT‐4 in Chinese, Korean, and Spanish clinical questions demonstrates that performance can vary substantially across languages, emphasizing the importance of multilingual validation and linguistic equity. Other studies examining AI‐generated abstracts, reviews, and informed‐consent documents similarly highlight both the opportunities and limitations of generative AI. Although AI‐generated text may be fluent and accessible, fluency does not necessarily equate to scientific rigor, citation accuracy, clinical completeness, or patient comprehension. These findings reinforce the continuing need for human oversight, fact‐checking, appropriate disclosure, and clear accountability when generative AI is incorporated into academic and clinical communication.

The Special Issue also demonstrates the expanding role of AI in diagnosis and disease characterization. Spatial pathology and spatial transcriptomics may provide new insights into the complex inflammatory and molecular heterogeneity of nasal polyps. Deep learning applied to multi‐sequence MRI and hyperspectral imaging shows the potential to extract information that may complement conventional clinical and pathological assessment. Similarly, AI‐based voice analysis may improve the detection and longitudinal monitoring of voice disorders by identifying subtle acoustic features. These approaches are promising, but many remain based on retrospective or single‐center datasets and require broader validation before routine clinical adoption.

Predictive AI provides another important direction. Prospective symptom‐based modeling for allergic rhinitis and machine‐learning prediction of patient‐reported outcomes in chronic rhinosinusitis illustrate how routinely collected clinical information may support earlier diagnosis and more individualized care. The study comparing LLM‐based and clinician prognostic reasoning in biologic‐treated chronic rhinosinusitis further suggests that generative AI may extract useful prognostic information from complex clinical narratives. Importantly, however, prediction is not synonymous with clinical benefit. The ultimate value of a predictive model depends on whether its output improves clinical decision‐making and, most importantly, patient outcomes.

AI is also beginning to enter the operating room. Automated surgical instrument tracking during endoscopic skull base surgery demonstrates the feasibility of using computer vision to characterize surgical activity and potentially provide objective information for education and assessment. Ambient AI documentation offers another potentially immediate application by reducing documentation burden and improving workflow efficiency. Yet these technologies introduce important considerations regarding accuracy, privacy, data governance, human factors, and responsibility for errors. Demonstrating that an AI system can recognize a surgical instrument or generate a clinical note is fundamentally different from demonstrating that it can safely improve patient care.

Perhaps the most important message emerging from this Special Issue concerns bias and equity. AI systems can inherit or amplify biases present in their training data, design, or clinical environment. The review of bias in otolaryngology and the experimental analysis of AI‐based prior authorization for head and neck reconstruction underscore the importance of evaluating AI performance across demographic and socioeconomic groups rather than assuming that an algorithm is inherently objective. Fairness, transparency, and continuous monitoring must therefore become integral components of AI development and implementation.

Taken together, these 15 contributions demonstrate both the extraordinary potential and the current limitations of AI in otolaryngology. Many studies remain retrospective, involve relatively limited datasets, or require external and prospective multicenter validation. Future research should increasingly focus not only on accuracy, but also on generalizability, uncertainty, subgroup performance, workflow integration, patient‐centered outcomes, and real‐world clinical effectiveness.

The central question for the next phase of AI research should therefore evolve from “Can AI perform this task?” to “For whom does it work, under what conditions, and does it improve patient care safely and equitably?” Human expertise will remain essential—not only for interpreting AI outputs, but also for defining clinically meaningful problems, recognizing limitations, incorporating patient preferences, and maintaining responsibility for clinical decisions.

This Special Issue reflects a field entering an important period of maturation. AI has the potential to enhance diagnosis, treatment, surgical education, documentation, communication, and personalized care throughout otolaryngology–head and neck surgery. Realizing that potential will require rigorous validation, scientific transparency, attention to equity, responsible implementation, and continued collaboration between clinicians and technology developers. The future of AI in our specialty should not be defined by whether machines can replace clinical expertise, but by how effectively they can augment human expertise and improve the care of our patients.

